# Dangshen Erling Decoction Ameliorates Myocardial Hypertrophy *via* Inhibiting Myocardial Inflammation

**DOI:** 10.3389/fphar.2021.725186

**Published:** 2022-01-03

**Authors:** Yigang Zhong, Liuying Chen, Miaofu Li, Lian Chen, Yufeng Qian, Chaofeng Chen, Yi Wang, Yizhou Xu

**Affiliations:** ^1^ Pharmaceutical Informatics Institute, College of Pharmaceutical Sciences, Zhejiang University, Hangzhou, China; ^2^ Department of Cardiology, Affiliated Hangzhou First People’s Hospital, Zhejiang University School of Medicine, Hangzhou, China; ^3^ Zhejiang Chinese Medical University, Hangzhou, China

**Keywords:** myocardial hypertrophy, heart failure, Dangshen Erling decoction, inflammation, TLR4 signaling pathway

## Abstract

Myocardial hypertrophy plays an essential role in the structural remodeling of the heart and the progression to heart failure (HF). There is an urgent need to understand the mechanisms underlying cardiac hypertrophy and to develop treatments for early intervention. Dangshen Erling decoction (DSELD) is a clinically used formula in Chinese medicine for treating coronary heart disease in patients with HF. However, the mechanism by which DSELD produces its cardioprotective effects remains largely unknown. This study explored the effects of DSELD on myocardial hypotrophy both *in vitro* and *in vivo*. *In vitro* studies indicated that DSELD significantly (p < 0.05) reduced the cross-sectional area of the myocardium and reduced elevated lactate dehydrogenase (LDH), tumor necrosis factor (TNF)-α, and interleukin (IL)-6 levels in the induced H9C2 cell model to study inflammation. *In vivo* experiments revealed that DSELD restores cardiac function and significantly reduces myocardial fibrosis in isoproterenol (ISO)-induced HF mouse model (p < 0.05). In addition, DSELD downregulated the expression of several inflammatory cytokines, such as granulocyte-macrophage colony-stimulating factor (GM-CSF), granulocyte CSF (G-CSF), IL-1α, IL-1β, IL-3, IL-5, IL-7, IL-12, IL-13, and TNF-α in HF (p < 0.05). Further analysis of the cardiac tissue demonstrated that DSELD produces its anti-inflammatory effects *via* the Toll-like receptor (TLR)4 signaling pathway. The expression of TLR4 downstream proteins such as matrix metalloproteinase-9 (MMP9) and myeloid differentiation factor-88 (MyD88) was among the regulated targets. In conclusion, these observations suggest that DSELD exerts antihypertrophic effects by alleviating the inflammatory injury *via* the TLR4 signaling pathway in HF and thus holds promising therapeutic potentials.

## Introduction

Heart failure (HF) is one of the most common forms of cardiac dysfunction caused by various cardiac diseases such as coronary heart disease, hypertension, arrhythmia, and viral myocarditis. HF is characterized by consistent pathological myocardial hypertrophy leading to increasing mortality and morbidity worldwide (Adamo et al., 2020). Pathological myocardial hypertrophy is characterized by enlarged cardiomyocytes and thickened ventricular walls that are the typical features of cardiac remodeling in HF (Tham et al., 2015). Initially, this adaptive change is to maintain the normal ejection fraction of the heart under increased pressure load; however, sustained pathological overload induces these changes to progress gradually into irreversible damage to the cardiac structure and function (Oka et al., 2014). Therefore, sustained myocardial hypertrophy is the key to HF. Recently, several studies suggest that persistent myocardial inflammation is the hallmark of myocardial hypertrophy (Geng et al., 2019; Zhang et al., 2020b; Zhu et al., 2020).

The inflammatory process in the myocardium manifests as a systemically chronic low-intensity reaction (Rogero and Calder, 2018). During this process, cardiomyocytes secrete various molecules including pro-inflammatory cytokines, colony-stimulating factor (CSF), and chemokines contributing to the infiltration of inflammatory cells into the myocardial interstitial tissue (Yang et al., 2016; Martini et al., 2019). In the sterile (non-infectious) inflammation of the myocardium, the pattern recognition receptor (PRR) is triggered by the damage-associated molecular patterns (DAMPs) such as endogenous tissue damage signaling molecules (Paul-Clark et al., 2012). Toll-like receptor (TLR)4, a member of the PRR family, has been most extensively studied due to its essential role in regulating cardiac inflammation (Dange et al., 2014; Deng et al., 2018). Current elucidation indicates that the development of myocardial hypertrophy is related to the TLR4 signaling pathway (Deng et al., 2018; Singh et al., 2019; Lu et al., 2020).

Traditional Chinese medicine (TCM), especially the formula that comprises several herbs containing complex compounds at specific ratios and doses, has been used effectively as alternative and complementary therapies in cardiovascular diseases in China and many other Asian countries, with a unique theoretical system (Jia et al., 2020). It has been widely applied to prevent and treat HF for more than 2,000 years (Hao et al., 2017; Liu et al., 2018). There is an increasing number of traditional herbal formulas such as Qili Qiangxin capsules that have been proven to be clinically effective for HF (Li et al., 2013). Dangshen Erling decoction (DSELD) is a traditional Chinese herbal formula with a clinical application of strengthening Qi, reinforcing the spleen, and promoting kidney function. It consists of six medicinal herbs, such as *Codonopsis pilosula* (Franch.) Nannf. (Dangshen), *Atractylodes macrocephala* Koidz. (Baizhu), *Smilax glabra* Roxb. (Fuling), *Saposhnikovia divaricata* (Turcz. ex Ledeb.) Schischk. (Fangfeng), *Gynochthodes officinalis* (F.C. How) Razafim. and B. Bremer (Bajitian), and *Glycyrrhiza glabra* L. (Gancao). Our previous study demonstrated that DSELD displayed various pharmacological effects, including antiapoptotic effects and mitochondrial protection (Zhong et al., 2020). High-performance liquid chromatography−mass spectrometry analysis for the structural components of the DSELD extract preliminarily identified 42 compounds, four of them have anti-inflammatory properties (tanshinone II, liquiritigenin, cimifugin, and scopoletin) (Zhong et al., 2020). Therefore, we speculated that DSELD suppresses inflammation to ameliorate myocardial hypertrophy in HF. However, further studies were needed to elucidate the underlying mechanism.

This study explores the effects and the underlying mechanism of DSELD in treating cardiac hypertrophy using *in vivo* and *in vitro* models. The results demonstrate that DSELD ameliorates myocardial hypertrophy *via* inhibiting the TLR4-mediated myocardial inflammation.

## Materials and Methods

### Chemicals and Reagents

The ingredients of DSELD described previously were provided by the Hangzhou First People’s Hospital (Hangzhou, China) (Zhong et al., 2020). Its composition is shown in Table 1. The herbal mixture was first extracted in water (1:8, w/v) twice by reflux for 2 h each time. After the extraction, the solutions were mixed, filtered, and concentrated. The final extract was concentrated to 0.83 g/ml of crude herb extract in water. The major components of DSELD were measured by liquid chromatography coupled with mass spectrometry to ensure consistency between batches (Supplementary Figure S1).

**TABLE 1:** The composition of Dangshen Erling decoction (DSELD).

Species	Herbal name	Part used	Dosage (g)	Ratio (%)
*Codonopsis pilosula* (Franch.) Nannf.	Dangshen	Root	30	36.14
*Smilax glabra* Roxb.	Fuling	Kernel	15	18.07
*Atractylodes macrocephala* Koidz.	Baizhu	Root	10	12.05
*Saposhnikovia divaricata* (Turcz. ex Ledeb.) Schischk.	Fangfeng	Root	10	12.05
*Gynochthodes officinalis* (F.C. How) Razafim. and B. Bremer	Bajitian	Root	10	12.05
*Glycyrrhiza glabra* L.	Gancao	Root	8	9.64
Total			83	100

Lipopolysaccharide (LPS; from *Escherichia coli* strain), isoprenaline hydrochloride [isoproterenol (ISO)], MTT assay kit, dimethyl sulfoxide (DMSO), and tetramethyl rhodamine methyl ester (TMRM) were purchased from Sigma Chemical Co. (St. Louis, MO, USA). Alexa Fluor 488 Phalloidin, anti-TLR4 (No. 14358), anti-nuclear factor (NF)-κB (No. 8242), anti-α-Tubulin (No. 3873), anti-β-actin (No. 4970) were purchased from Cell Signaling Technology (Danvers, MA, USA). Anti-matrix metalloproteinase (MMP)9 (No. ab38898) and anti-myeloid differentiation factor (MyD)88 (No. ab 2064) were purchased from Abcam (Cambridge, MA, USA). The nitric oxide (NO) assay and lactate dehydrogenase (LDH) cytotoxicity assay kits were purchased from Beyotime Biotechnology (Shanghai, China). The tumor necrosis factor (TNF)-α ELISA kit, interleukin (IL)-6 ELISA kit, and bicinchoninic acid (BCA) assay kit were purchased from Thermo Fisher Scientific (Eugene, OR, USA). The NT-proBNP ELISA kit was purchased from Yubo Biotechnology (Shanghai, China). Assays were run based on manufacturer’s instructions.

### Isoproterenol-Induced Cardiac Hypertrophy Model in Mice

A total of 60 male C57/BL6 mice each weighing more than 20 g and between 8 and 10 weeks of age with the specific pathogen-free (SPF) grade were purchased from Shanghai Slac Laboratory Animal Co., Ltd. (Shanghai, China). Mice were randomly divided into the vehicle (saline) group (*n* = 20) and the ISO group (*n* = 40). Each mouse received either 5 mg/kg/day ISO (ISO group) or saline (Vehicle group) by subcutaneous injection for 4 weeks. At the same time, half of the mice in the ISO group were randomly selected for DSELD treatment (*n* = 20). The treatment group received a gavage of DSELD at a dosage of 1.28 g (crude herbs)/kg (calculated based on body surface area) for 4 weeks. Transthoracic echocardiography was performed to evaluate the heart function at the end of the fourth week. Afterward, the blood was collected for the detection of the NT-proBNP and inflammatory cytokines. Subsequently, the animals were sacrificed by cervical dislocation, and the hearts were collected for further examination.

### Echocardiography

Isoflurane (inhaled)-anesthetized mice were subjected to 2-dimensional M-mode and B-mode echocardiography (Vevo TM 2100; Visual Sonics, Canada) to evaluate cardiac function. The ejection fraction (EF) and fractional shortening (FS) were measured as previously reported (Zhang et al., 2020b).

### Histological Examination and Immunofluorescence Analysis

The heart was cut into 5-μm cross-sections to analyze the cardiac structure by hematoxylin–eosin (H&E) staining and fibrosis by Sirius Red staining. All stained sections were observed under an inverted microscope. Image Pro Plus 6.0 software (Media Cybernetics, Rockville, MD, USA) was used to map and analyze the area of fibrosis of the Sirius Red staining cross-sections by the software mapping module.

For immunofluorescence staining, cardiac tissues were frozen sectioned into 30-µm slices and blocked with 5% bovine serum albumin (BSA) for 0.5 h. After washing, the tissue sections were incubated with fluorescently labeled MPP9 antibody (1:250), MyD88 antibody (1:250), and NF-κB antibody (1:250). Image Pro Plus 6.0 software (Media Cybernetics) was used to analyze the fluorescence intensity.

### Detection of Inflammatory Cytokine Profile in Cardiac Hypertrophy Model

About 50–100 mg myocardial tissue from different experimental groups were collected to perform multiple cytokine assays by Hangzhou AiTing Biological Technology Co., Ltd. (Hangzhou, China).

### Measuring Nitric Oxide in RAW264.7 Macrophage Culture

The RAW264.7 macrophages used in this study were obtained from the Cell Bank of Type Culture Collection of the Chinese Academy of Sciences (Shanghai, China). The RAW264.7 macrophages were cultured in Dulbecco’s modified Eagle’s medium (DMEM) with 10% fetal bovine serum (FBS; pre-inactivated at 56°C for 30 min), penicillin (100 U/ml), and streptomycin (100 μg/ml) at 37°C under 5% CO_2_ and 95% air. The effects of DSELD on LPS-activated RAW264.7 macrophages were evaluated after cells were exposed to (1 μg/ml) LPS for 24 h with or without DSELD (Zhang et al., 2018). DSELD at 50 μg/ml was administered to treatment groups as previously described (Zhong et al., 2020). The cell supernatants were collected and stored at -20°C to measure NO concentration using a commercial ELISA kit.

### Measuring Lactate Dehydrogenase, Tumor Necrosis Factor-α, and Interleukin-6 in H9C2 Cell Culture

The H9C2 cells used in this study were obtained from Nanjing Beretti Biological Technology Co., Ltd. (Nanjing, China), and cultured in DMEM with 10% FBS, penicillin (100 U/ml), and streptomycin (100 μg/ml) at 37°C, 5% CO_2_ and 95% air. The macrophage-conditioned media (CM) stimulation model was conducted as previously described (Li et al., 2016). To evaluate the effects of DSELD on the CM-stimulated H9C2 cells, the H9C2 cells were incubated with CM and treated with DSELD at 50 μg/ml for 24 h. The cell supernatants were collected for detection of LDH, TNF-α, and IL-6.

### Measurement of Cell Viability

MTT assay was used to assess cell viability. The H9C2 cells were seeded onto 96-well plates at a density of 5 × 10^4^/well. Then, 100 μl of MTT (5 mg/ml) was added to each well for 4 h. The optical density (OD) was measured at a wavelength of 580 nm after adding DMSO.

### High-Content Screening Assay

H9C2 cell monolayers were incubated with 100 nM TMRM for 45 min at 37°C to monitor the TMRM fluorescence (Branco et al., 2013). Cell monolayers were then fixed with cold 4% paraformaldehyde for 30 min, followed by 1% Triton X-100 treatment for 10 min. After three washes with phosphate buffered saline (PBS), the cells were incubated in a mixture of Alexa Fluor 488 Phalloidin (1:20) and Hoechst (1:1,000) at room temperature for 10 min (Guo et al., 2020). The ImageXpress Micro® Confocal High-Content Imaging System (Molecular Devices, LLC, San Jose, CA, USA) was used to acquire images. The analysis module of the MetaXpress® High-Content Image and Analysis Software (Molecular Devices) was used to analyze the images. The cell cross-sectional area was normalized to the nucleus count.

### Western Blot Analysis

Cardiac tissues were lysed using radioimmunoprecipitation assay (RIPA) buffer [50 mM Tris–HCl pH 7.4, 150 mM NaCl, 1% NP-40, and 0.1% sodium dodecyl sulfate (SDS)] containing a protease inhibitor cocktail (Sigma, St. Louis, MO, USA). H9C2 cells were digested by trypsin, and the collected cells were prepared for cell lysis, and proteins were extracted according to the manufacturer’s instruction. Western blotting was performed to detect the levels of various proteins in heart tissue or H9C2 cell lysates quantified by BCA assay. A 12% SDS polyacrylamide gel electrophoresis (SDS-PAGE) was used to separate the proteins. Then, the proteins were transferred to polyvinylidene fluoride (PVDF) membrane (Millipore, Burlington, MA, USA). After blocking with 10% skim milk, the membranes were incubated with primary antibodies (TLR4 1:1,000; NF-κB 1:1,000; α-tubulin 1:1,000; β-actin 1:1,000; MMP9 1:1,000; MyD88 1:1,000) at 4°C overnight. After incubation with the appropriate secondary antibodies at room temperature for 1 h, signals were visualized using the enhanced chemiluminescence (ECL) Plus Western blotting detection reagents (Bio-Rad) for 1 min at room temperature. The bands in the membrane were visualized, and densitometric analysis of band intensity was performed using the ChemiDoc Touch Imaging System and Image Lab software (Bio-Rad, Hercules, CA, USA).

### Statistical Analysis

The continuous variables were shown as mean ± SD. Statistical analyses were performed using one-way analysis (ANOVA) of variance. Tukey’s and Dunnett’s tests were applied for multiple comparisons between groups. GraphPad Prism seven software (GraphPad Software, San Diego, CA, USA) was used to carry out the statistical analysis. p < 0.05 was considered statistically significant.

## Results

### Dangshen Erling Decoction Attenuated Cardiac Hypertrophy in the Mouse Model of Heart Failure

**FIGURE 1:**
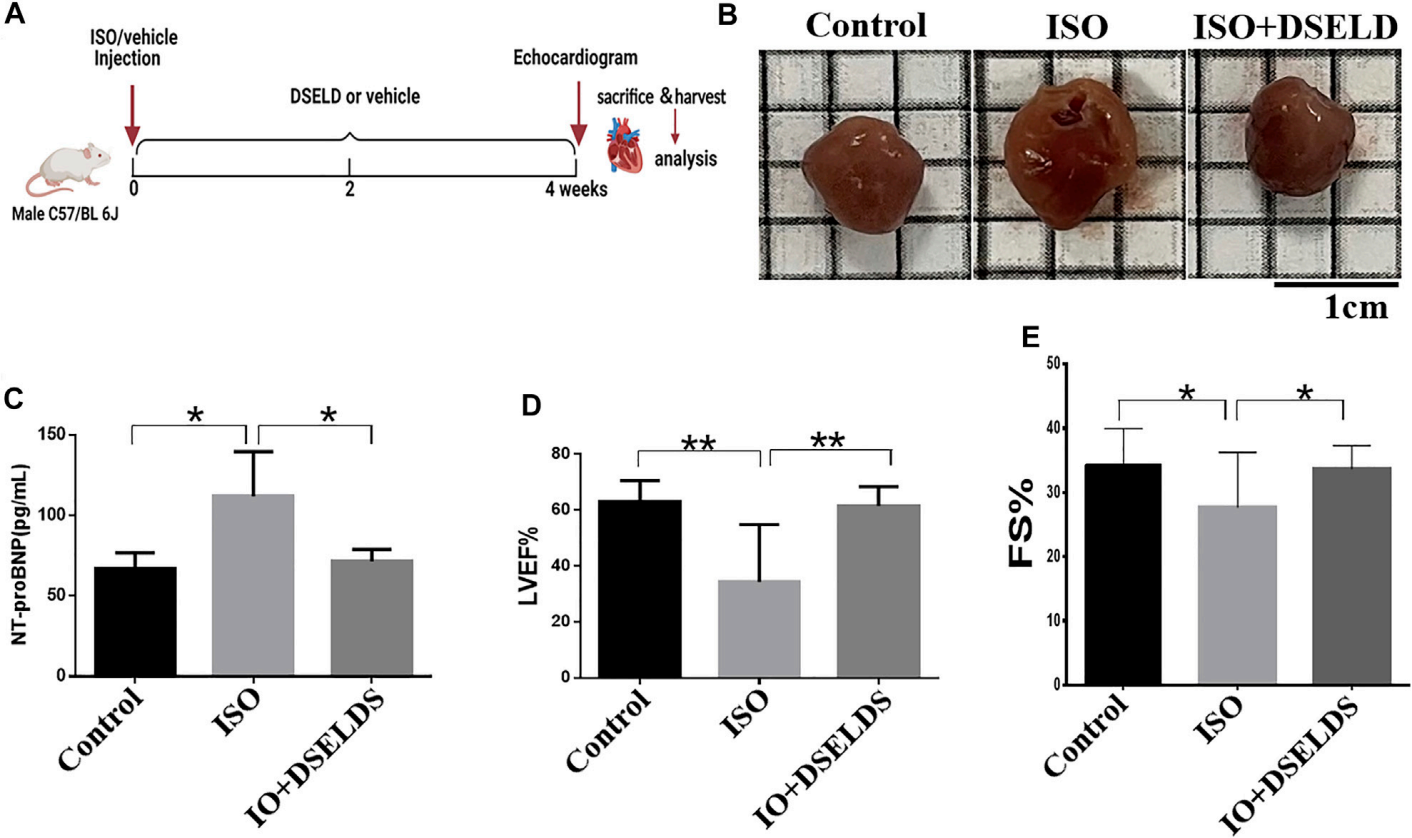
Dangshen Erling decoction (DSELD) improves cardiac function. **(A)** Establishment of heart failure (HF) mouse model. **(B)** The representative images of the heart from the isoproterenol (ISO)-induced HF mice with or without DSELD (1.28 g/kg) treatment. (**C–E**) NT-proBNP, left ventricular ejection fraction (LVEF), and fractional shortening (FS) in the ISO-induced HF mice with or without DSELD (1.28 g/kg) treatment, n = 9–10. *p < 0.05, **p < 0.01.

To evaluate the effects of DSELD on cardiac hypertrophy, DSELD was intragastrically administered at a dose of 1.28 g/kg in the ISO-induced mouse model for 4 weeks (Figure 1A). The ISO-treated mice showed enlarged hearts compared to the control mice **(** Figure 1B **)**. Moreover, the NT-proBNP, an important indicator of HF, was significantly upregulated compared with the control group (Figure 1C). Echocardiography revealed that left ventricular ejection fraction (LVEF) and fractional shortening (FS) decreased in the ISO group (Figures 1D, E), indicating that heart functions were severely damaged in this group. After DSELD administration for 4 weeks, the size of the enlarged heart decreased, and the NT-proBNP, EF, and FS of the treated group were significantly restored. These investigations showed that DSELD exhibited cardioprotective properties in HF and almost completely recovered heart function.

**FIGURE 2:**
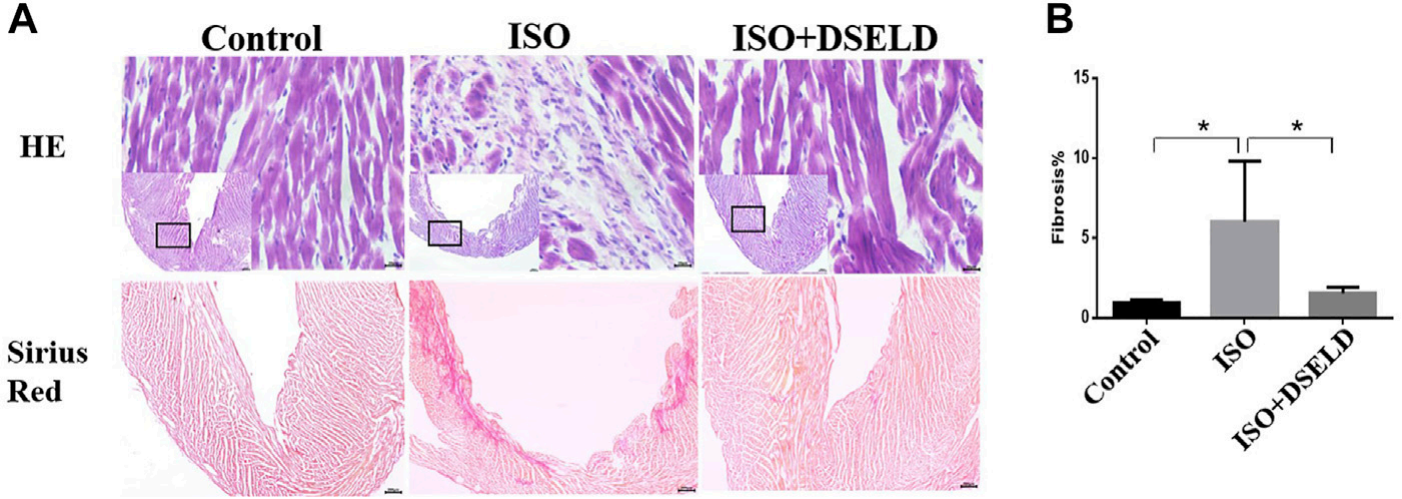
Dangshen Erling decoction (DSELD) attenuates ventricular remodeling. **(A)** Typical histological images of the mouse cardiac tissue from the different groups were stained with H&E (×400) and Sirius Red (×40). **(B)** Quantification of cardiac fibrosis in the different groups of mice, n = 4. *p < 0.05, **p < 0.01.

Cardiac tissue morphology by H&E and Sirius Red staining was examined using microscopy. The structures of the left ventricle in the ISO-treated group showed large necrotic areas obvious by H&E staining **(** Figure 2A **)**. Furthermore, the adjacent cardiomyocytes were arranged randomly. Sirius Red staining revealed that the total cardiac fibrosis area in the ISO-induced group was enlarged by approximately 5-fold compared to that of the control group (Figure 2B **)**. However, DSELD treatment significantly suppressed the pathological changes including myocardial remodeling and fibrosis caused by ISO. These results proved that DSELD could prevent the ISO-induced damages of the cardiac structure and the proliferation of collagen fibers.

### Dangshen Erling Decoction Alleviated the Conditioned Media-Induced Myocardial Hypertrophy in H9C2 Cells

**FIGURE 3:**
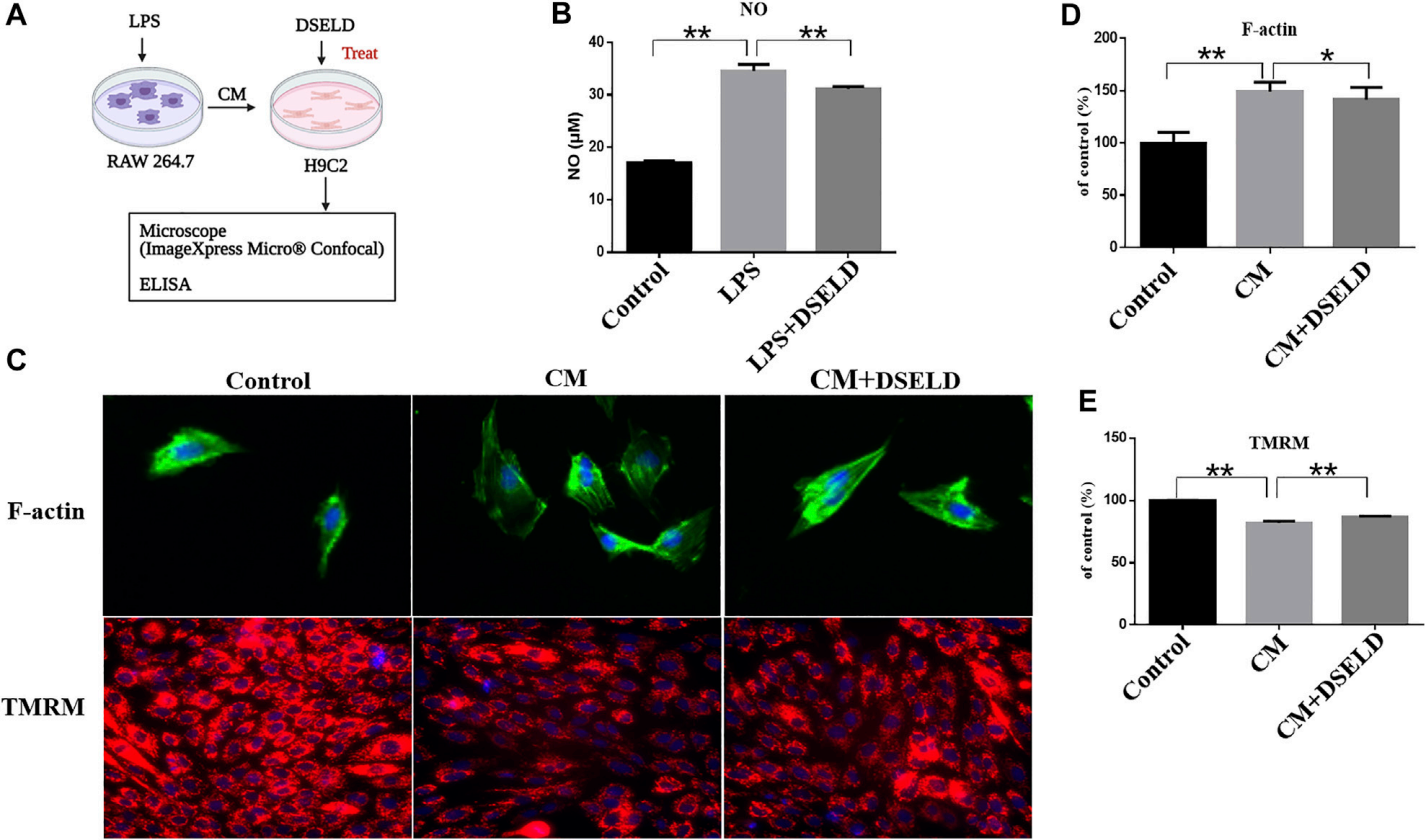
Dangshen Erling decoction (DSELD) protects from myocardial hypertrophy and mitochondrial permeability transition pore (mPTP) in the conditioned media (CM)-induced inflammation in H9C2 cells. **(A)** The operation mode diagram of the RAW264.7 and H9C2 cells. **(B)** RAW264.7 cells were incubated with lipopolysaccharide (LPS; 1 μg/ml) with or without DSELD (50 μg/ml) for 24 h, and nitric oxide (NO) secretion was detected in cell supernatant by ELISA. **(C)** Representative fluorescent images of cardiomyocytes from the different groups [green: phalloidin, red: tetramethyl rhodamine methyl ester (TMRM), and blue: DAPI]. **(D)** The relative area was corrected by the nucleus and normalized to control (F-actin, ×400). **(E)** The fluorescence intensity to control (TMRM, ×200). *p < 0.05, **p < 0.01.

To further investigate the effect of DSELD in ameliorating myocardial hypertrophy, we next established the myocardial hypertrophy model of macrophage-CM-stimulated H9C2 cells (Li et al., 2016; Zhang et al., 2018) (Figure 3A). The RAW264.7 macrophage cells were stimulated with 1 μg/ml LPS. After LPS stimulation, RAW264.7 macrophages secreted nearly twice the amount of NO as that of the control DMEM-treated cells (Figure 3B), affirming that LPS stimulation of RAW264.7 macrophages was effective. DSELD treatment reduced NO levels, thus inhibiting the effect of LPS stimulation on RAW264.7 macrophages. Then, the supernatants were obtained from the RAW264.7 cells after LPS stimulation for 24 h to imitate the inflammatory environment, as performed for H9C2 cells stimulated by CM (Zhang et al., 2018). The antihypertrophy effects of DSELD (50 μg/ml) on cardiac cells were investigated while establishing the CM-induced myocardial hypertrophy model.

High-Content Imaging System assay was used to evaluate the beneficial effect of DSELD on myocardial hypertrophy and mitochondrial permeability transition pore (mPTP) in the CM-stimulated myocardial hypertrophy model. Evaluation was done with or without DSELD (50 μg/ml) treatment. As shown in Figures 3C and D, the average of cardiomyocyte cross-sectional area after CM stimulation significantly enlarged by approximately 50% in comparison to that of the control group. This result showed the successful establishment of the CM-stimulated hypertrophic cardiomyocyte model. However, DSELD treatment reduced the size of CM-induced cardiomyocytes, suggesting the critical role of DSELD in protecting against CM-induced myocardial hypertrophy. Moreover, CM-induced cardiomyocytes exhibited an inferior TMRM fluorescence ability that inversely correlated to the opening of mPTP. Additionally, DSELD significantly potentiated TMRM fluorescence ability in the CM-stimulated H9C2 cell model, demonstrating a decrease in mPTP opening that protected against CM-induced damages in the mitochondrial membrane permeability (Figures 3C, E). These results indicate that CM-induced inflammation promotes myocardial hypertrophy as well as the opening of the mPTP, while DSELD alleviates myocardial hypertrophy and restores the mPTP damage in the CM-stimulated H9C2 cells.

### Dangshen Erling Decoction Alleviates Myocardial Injury Induced by Conditioned Media

**FIGURE 4:**
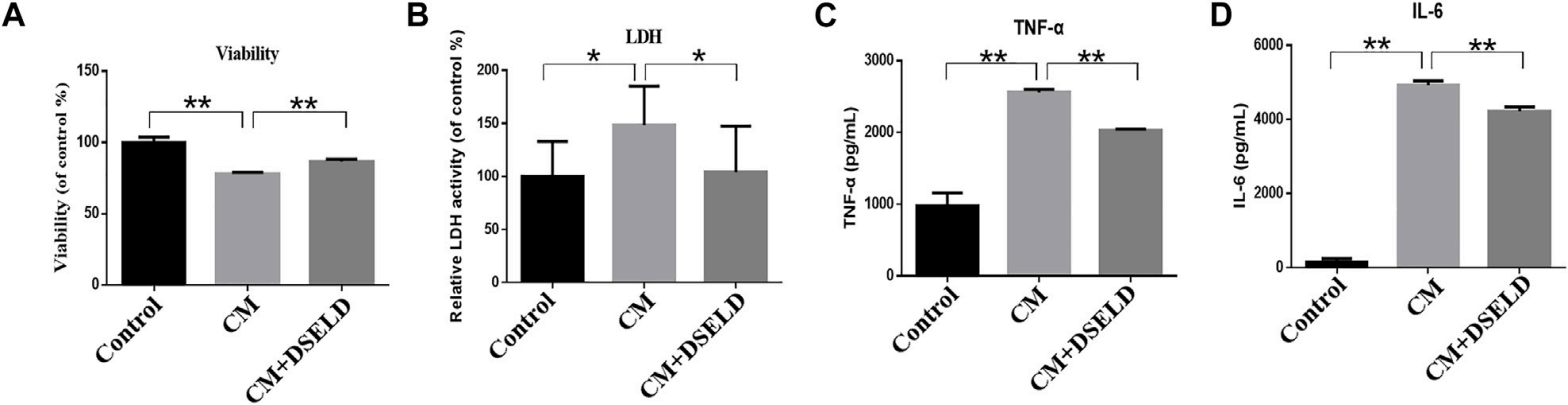
Dangshen Erling decoction (DSELD) inhibits conditioned media (CM)-induced inflammation in the H9C2 cells. **(A)** H9C2 cell viability was evaluated by MTT assay 24 h after incubation with CM. **(B–D)** The elevation of lactate dehydrogenase (LDH), tumor necrosis factor (TNF)-α, and interleukin (IL)-6 from different groups. *p < 0.05, **p < 0.01.

To confirm the antihypertrophy effect of DSELD on inflamed cardiomyocytes, inflammatory markers were evaluated in hypertrophied H9C2 cells. The results show that CM aggravated H9C2 cell death, while DSELD treatment protected cell viability **(** Figure 4A **)**. The levels of inflammatory markers including LDH, TNF-α, and IL-6 were significantly elevated after CM induction but were suppressed by DSELD treatment (Figures 4B–D **)**. Consistently, the myocardial injury induced by CM was obvious. This damage was related to the inflammatory microenvironment of cardiomyocytes that could be rescued by DSELD treatment.

### Dangshen Erling Decoction Downregulates the Multiple Inflammatory Cytokines in the Mouse Model of Cardiac Failure

**FIGURE 5:**
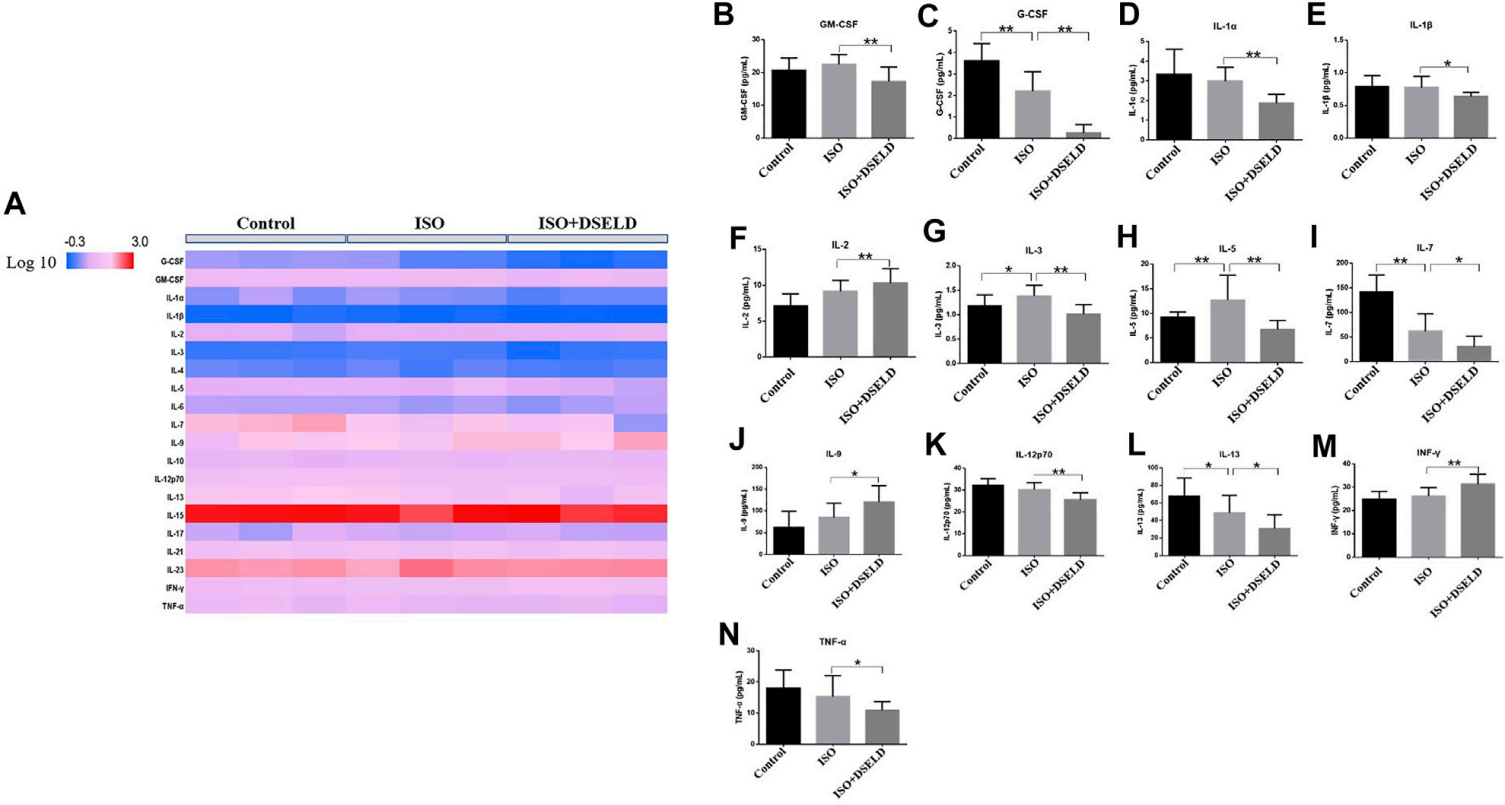
Multiple inflammatory cytokines in heart failure (HF). **(A)** Cytokine profiling of heart samples obtained from the different treatment groups. **(B–N)** Cytokine concentrations in heart samples from different treatment groups of mice, n = 4. *p < 0.05, **p < 0.01.

To further confirm the anti-inflammatory effect of DSELD *in vivo*, multiple inflammatory cytokines were evaluated in the cardiac tissue (Figure 5A). The data show that granulocyte-macrophage CSF (GM-CSF), granulocyte CSF (G-CSF), IL-1α, IL-1β, IL-3, IL-5, IL-7, IL-12, IL-13, and TNF-α decreased significantly in the treated group vs. the ISO group **(** Figures 5B–N **)**. However, some other inflammatory cytokines such as IL-2, IL-9, and interferon (INF)-γ were elevated in DSELD group vs. the ISO group. Notably, increased levels of some cytokines such as IL-1α, IL-1β, and TNF-α have been reported previously in HF compared with controls and were suggested as markers of inflammatory progression in HF (Vistnes et al., 2010). The results of this study indicate that DSELD exhibits an inhibitory effect on multiple inflammatory cytokines in the myocardial tissue.

### Dangshen Erling Decoction Alleviates Myocardial Hypertrophy by Inhibiting the Toll-Like Receptor 4 Signaling Pathway

**FIGURE 6:**
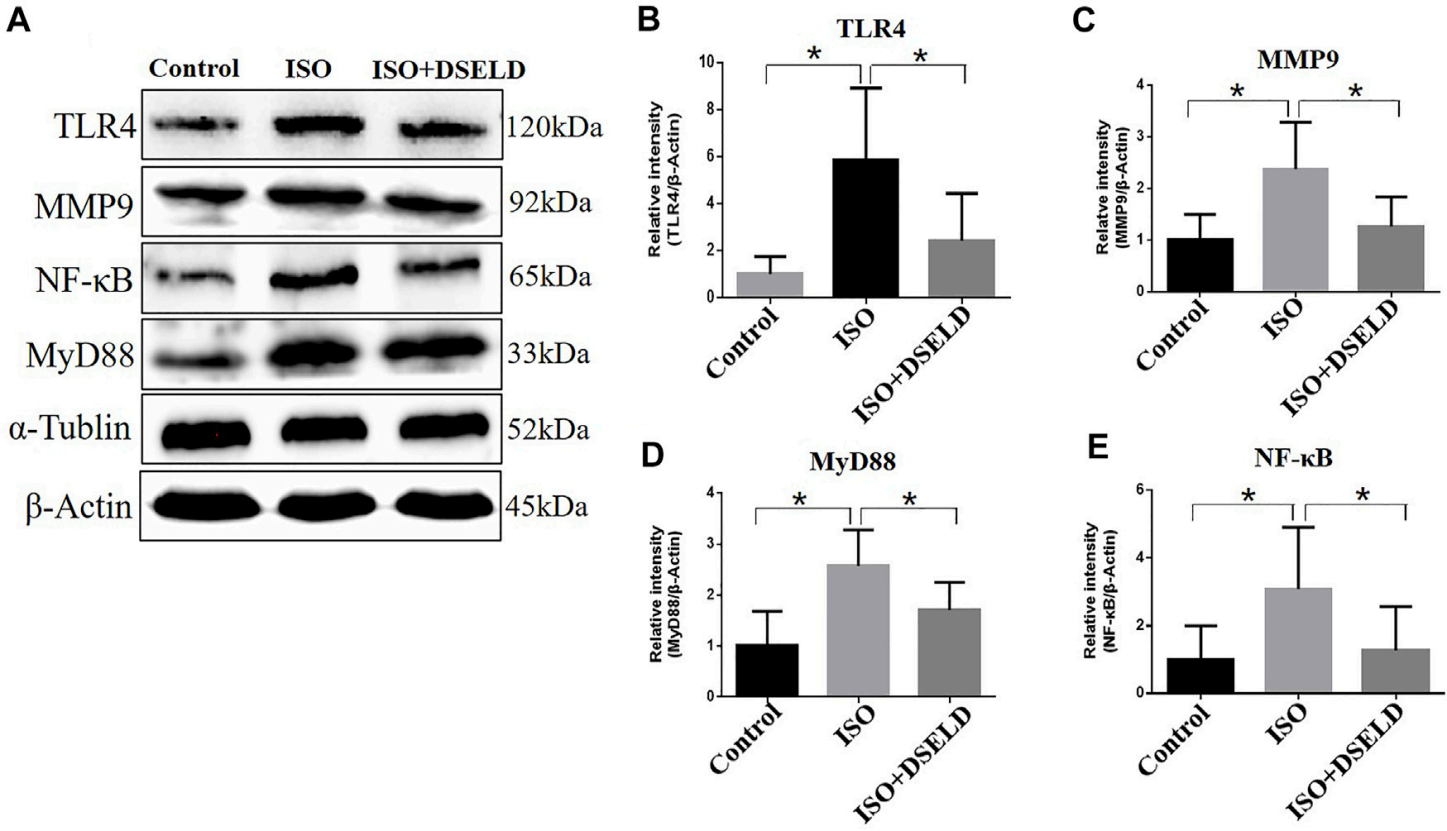
Dangshen Erling decoction (DSELD) inhibits the expressions of Toll-like receptor (TLR)4 signaling pathway proteins. **(A)** Western blot of TLR4, matrix metalloproteinase (MMP)9, nuclear factor (NF)-κB, and myeloid differentiation factor (MyD)88 proteins and **(B–E)** relative expression in the heart samples normalized to control. n = 4. *p < 0.05, **p < 0.01.

Previous studies suggest that TLR4 signaling pathway plays a critical role in the release of inflammatory cytokines (Zhang et al., 2020a). Therefore, we hypothesized that DSELD protects the cardiomyocytes from inflammation by inhibiting the TLR4 signaling pathway. To verify this notion, further investigation into the changes of key molecules in this signaling pathway with or without DSELD treatment was done, and the expressions of the TLR4, MyD88, and MMP9 in the cardiac tissue were detected (Wang et al., 2016; Wang et al., 2019). Western blot analysis revealed an increase in the protein levels of TLR4, MMP9, MyD88, and NF-κB in the ISO-induced group (Figure 6). However, DSELD treatment significantly inhibited the levels of these proteins, indicating that DSELD regulates inflammation by inhibiting the TLR4 signaling pathway.

**FIGURE 7:**
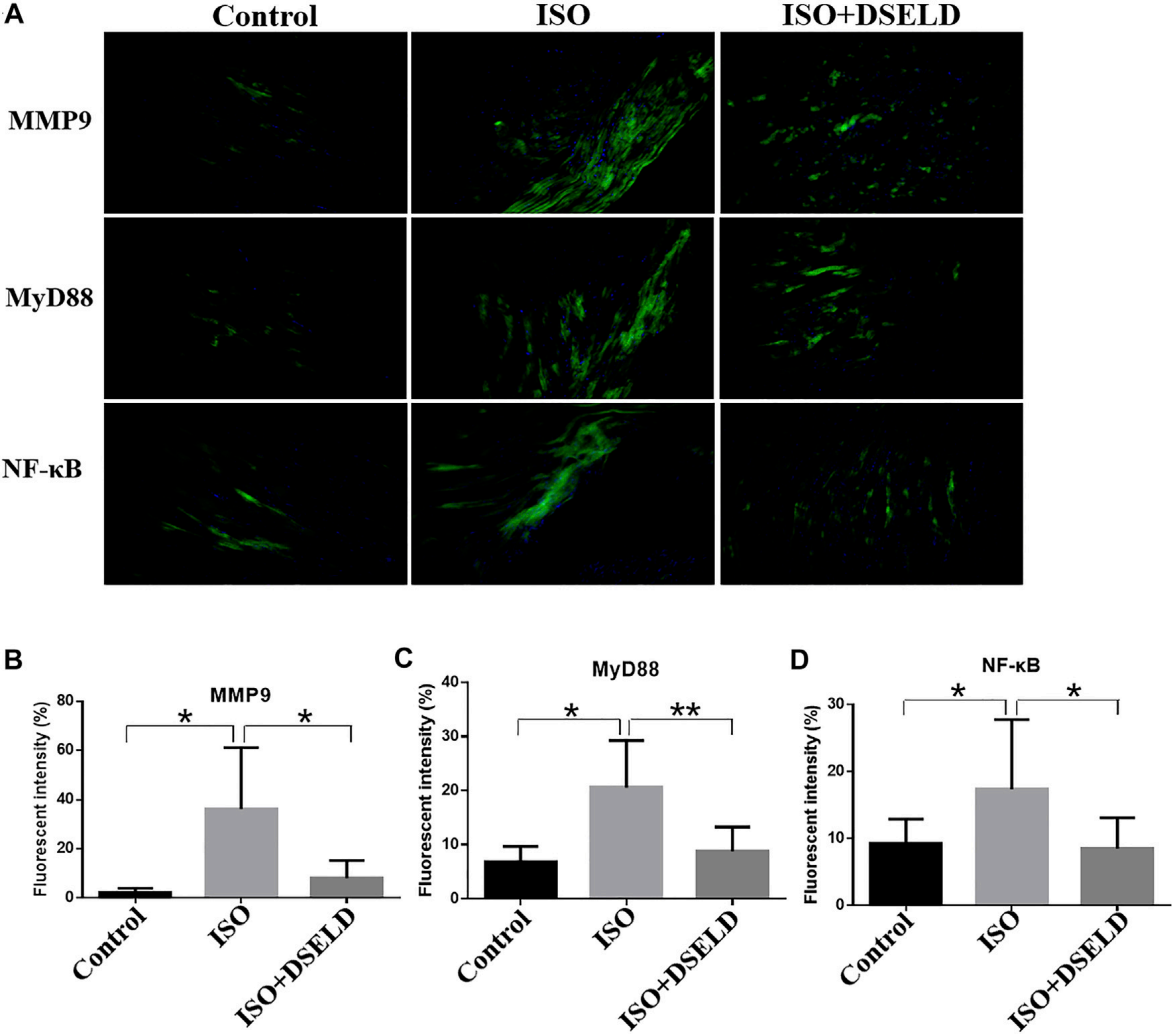
Dangshen Erling decoction (DSELD) attenuates cardiac inflammation by inhibiting the expression of members in the Toll-like receptor (TLR)4 signaling pathway in the cardiac tissue. **(A)** Representative immunofluorescence images of the heart samples from different treatment groups [green: matrix metalloproteinase (MMP)9, myeloid differentiation factor (MyD)88, and nuclear factor (NF)-κB proteins; blue: DAPI, ×200]. **(B–D)** Quantification of the relative fluorescence intensity from different groups of mice, n = 4. *p < 0.05, **p < 0.01.

*In situ* immunofluorescence evaluation revealed similar results (Figure 7). The fluorescence of MMP9, MyD88, and NF-κB in the heart was significantly potentiated, demonstrating the upregulated expressions of these proteins in the ISO-induced myocardial tissue compared to the control group. Furthermore, the fluorescence intensity decreased significantly after DSELD treatment. It also proved that DSELD suppresses the inflammatory process in myocardial hypertrophy by regulating the expressions of these key proteins in the TLR4 signaling pathway. Taken together, the results indicate that DSELD alleviates myocardial hypertrophy by inhibiting the expressions of TLR4 signaling pathway-associated proteins.

## Discussion

Our previous study shows that the chemical composition of DSELD has anti-inflammatory properties and evaluates its cardioprotective effects and antiapoptotic potentials in H9C2 cells (Zhong et al., 2020). In this work, comprehensive *in vitro* and *in vivo* experiments are conducted to investigate the mechanisms by which DSELD exerts its antihypertrophic properties. The main findings of this study are as follows: 1) DSELD protects H9C2 cells from CM-induced injuries; 2) DSELD restores the cardiac functions and reverses the pathological damages in a mouse model of myocardial hypertrophy; 3) DSELD modulates the levels of multiple inflammatory cytokines; 4) DSELD inhibits inflammation by downregulating the expression of inflammatory proteins such as MyD88 and MMP9, key components of the TLR4 signaling pathway.

Cardiac hypertrophy in the adult heart is manifested as an increase in the size instead of the number of cardiomyocytes obviously because the cardiomyocytes cannot proliferate after birth (Nakamura and Sadoshima, 2018). In our work, the macrophage-CM stimulation was used to establish a successful *in vitro* cardiac hypertrophy model. The cross-sectional area of cardiomyocytes enlarged after LPS-triggered CM stimulation (Figures 3C, D). The results also show that CM stimulation elevates the levels of LDH, TNF-α, and IL-6. Infiltration of these inflammatory mediators results in myocardial hypertrophy and fibrosis followed by progression into ventricular remodeling (Ismahil et al., 2014; Shimizu and Minamino, 2016; Bacmeister et al., 2019).

In addition, this study demonstrates that CM stimulation affects the opening of mPTP detected by TMRM staining (Figures 3C, E). The weak fluorescence intensity indicates prolonged opening of mPTP. Prolonged mPTP opening leads to mitochondrial energy metabolism disorder, destruction of organelle structure, and typical necrotic cell death, which finally leads to cardiac dysfunction (Kwong and Molkentin, 2015). In our study, the weakest fluorescence intensity of the TMRM was observed in the CM-induced H9C2 cell model, indicating that mPTP is also damaged by inflammation after CM treatment.

Many recent studies suggest that inflammation plays an essential role in promoting HF, and the progression of pathological cardiac remodeling is dependent partly on autoimmune injuries of the heart (Burchfield et al., 2013; Ismahil et al., 2014; Adamo et al., 2020). In this study, ISO-induced myocardial hypertrophy mouse model was used as an *in vivo* model to evaluate DSELD cardioprotective potentials. In addition to monitoring NT-proBNP, EF, FS, and heart structure, multiple cytokine assays were performed on cardiac tissue instead of serum to evaluate the levels of multiple inflammatory cytokines. The results of these assays indicate that multiple inflammatory cytokines such as GM-CSF, G-CSF, IL-1α, IL-1β, IL-3, IL-5, IL-7, IL-12, IL-13, and TNF-α were significantly decreased in the DSELD group vs. the ISO group (Figure 5). It has been reported that these cytokines are associated with the activation of CD4^+^ T cells, which play a pro-inflammatory role during chronic inflammation of HF (Quast et al., 2017). Interestingly, among these inflammatory cytokines, the levels of the G-CSF, IL-7, and IL-13 decreased following ISO treatment. The activation of CD4^+^ T cells in the myocardial inflammatory microenvironment may lead not only to an increase of chronic inflammatory cytokines but also to an increase of other anti-inflammatory cytokines like IL-17A, which potentially counteracts the pro-inflammatory cytokines, such as G-CSF (Dick and Epelman, 2016). This regulation may be closely related to the timing of cardiac inflammation. For example, the expression level of G-CSF increases rapidly after myocardial ischemia–reperfusion injury (acute inflammation), peaks at day 1, and decreases thereafter (Fan et al., 2019). As inflammation becomes chronic, IL-17A starts to gradually increase to further reduce the G-CSF and suppress myocardial inflammation. IL-7 is not only an inflammatory cytokine but also a hematopoietic growth factor secreted by bone marrow stromal cells whose expression is regulated by the hematopoietic ability of bone marrow (Nguyen et al., 2017). Bone marrow dysfunction is a poor complication of HF leading to the deficiency of hematopoietic growth factor including IL-7 (Westenbrink et al., 2010; Ruifrok et al., 2011). In our study, ISO-induced HF may be associated with the decreased IL-7 levels. Meanwhile, insufficient IL-7 may lead to the dysfunction of innate lymphocyte cell, which collectively lead to impair the cytokines it secretes such as IL-13 (Soong et al., 2014). Furthermore, multiple CD4^+^ T cell-associated cytokines were analyzed as dynamic profiles at different stages of HF development (Wang et al., 2018; Yuan et al., 2019). Moreover, studies have shown that the activation of CD4^+^ T cells after trauma was TLR4-dependent (Bock et al., 2018). Therefore, we hypothesized that the regulation of these cytokines is associated with the TLR4 signaling pathway (Shi et al., 2006; Gitlin et al., 2020). We further verified the expression of TLR4 signaling pathway molecules with or without DSELD in the myocardial hypertrophy model. The *in vivo* study results show that DSELD not only promoted heart functions and decreased the levels of multiple cytokines but also inhibited the expression of TLR4 pathway-associated proteins.

The TLR4 signal pathway plays an essential role in inflammation (Andersson and Tracey, 2011). TLR4 is the first mammalian Toll protein in the TLR family to be characterized. It is expressed in immune-related cells, including monocytes, macrophages, dendritic cells, as well as adipocytes, enterocytes, and muscle cells to control the inflammatory and immunological responses (Kuzmich et al., 2017). Under stimulation by inflammation-causing substances like LPS, TLR4 induces the production of multiple cytokines including TNF-α and IL-1β, which, in turn, work as endogenous inflammatory inducers by interacting with receptors on target cells (Lancaster et al., 2018; Rogero and Calder, 2018). Several studies revealed that TLR4 is a pivotal modulator of myocardial inflammation, and the high expression of TLR4 is a risk factor for HF (Frantz et al., 1999; Földes et al., 2008; Liu et al., 2015; Rogero and Calder, 2018).

The mechanism by which TLR4 influences myocardial hypertrophy mainly involves the MyD88-dependent pathway and MMP9-dependent pathway (Yang et al., 2016). The MyD88-dependent pathway is initiated after TLR4 activation, thus inducing many transcription factors such as NF-κB. The activation of NF-κB contributes to the expression of inflammatory cytokines like IL-6 and TNF-α (Xu et al., 2020). Supportively, our results demonstrated the high expression of MyD88, NF-κB, and MMP9 in mice with myocardial hypertrophy. Importantly, DSELD treatment significantly downregulates the expressions of MyD88, NF-κB, and MMP9, revealing that the antihypertrophic effects of the drug may work through suppressing the activated TLR4 pathway.

## Conclusion

In summary, our study demonstrates that DSELD could protect against myocardial hypertrophy by inhibiting myocardial inflammation. The antihypertrophic mechanism of DSELD might be mediated by the suppression of the TLR4 signaling pathway. But further study is needed to determine which compound in the formula is the major active component. Our findings provide new insight to further understand the pharmacological mechanism of DSELD and bring new therapeutic candidates in the management of HF.

## Data Availability

The original contributions presented in the study are included in the article/Supplementary Material. Further inquiries can be directed to the corresponding authors.
