# Supplementary material for: Dangshen Erling Decoction Ameliorates Myocardial Hypertrophy via Inhibiting Myocardial Inflammation
Source: Front Pharmacol. 2022 Jan 3;12:725186. doi: 10.3389/fphar.2021.725186 (PMC8762257; doi:10.3389/fphar.2021.725186)
Supplement: Supplementary file 1 [file Image1.pdf]

Supplementary Material

Supplementary Figure

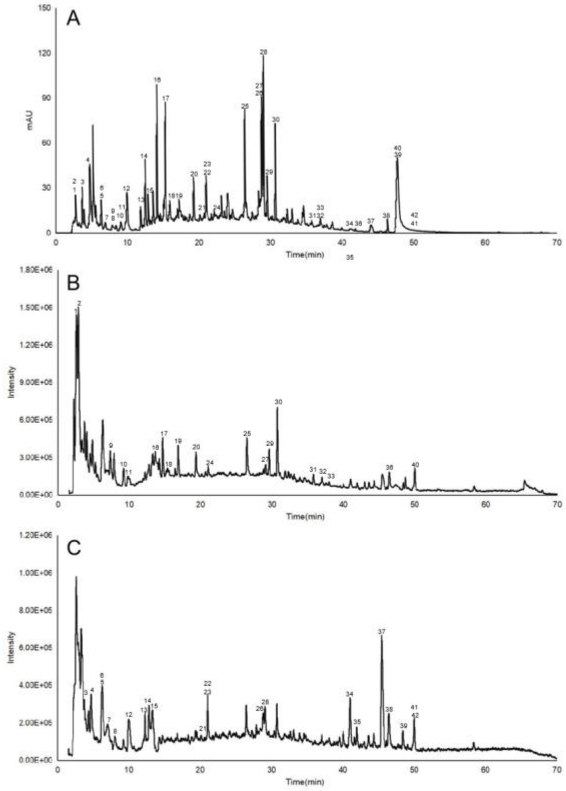

Supplementary Figure 1. Total ion chromatogram (TIC)（A）in positive mode (B) and negative mode (C) of DSELD.
